# Effect of Nonalcoholic Fatty Liver Disease and Metabolic Risk Factors on Waitlist Outcomes in Patients With Hepatocellular Carcinoma

**DOI:** 10.1097/TXD.0000000000001060

**Published:** 2020-09-17

**Authors:** Kelley Weinfurtner, Jennifer L. Dodge, Francis Y. K. Yao, Neil Mehta

**Affiliations:** 1 Department of Medicine, University of California, San Francisco, San Francisco, CA.; 2 Division of Gastroenterology and Hepatology, Department of Medicine, University of Pennsylvania, Philadelphia, PA.; 3 Division of Gastroenterology, Department of Medicine, University of California, San Francisco, San Francisco, CA.; 4 Division of Transplant Surgery, Department of Surgery, University of California, San Francisco, San Francisco, CA.

## Abstract

Nonalcoholic fatty liver disease (NAFLD) is a leading cause of hepatocellular carcinoma (HCC) in the United States. Prior data suggest that NAFLD-HCC patients are less likely to receive liver transplantation (LT) and have worse overall survival; however, the reason for this discrepancy is unknown.

**Methods.:**

We conducted a retrospective study of 631 HCC patients listed for LT at a large academic center from 2004 to 2013. Waitlist dropout and LT were analyzed using competing risk regression.

**Results.:**

Compared with other-HCC patients (n = 589), NAFLD-HCC patients (n = 42, 6.7%) were older (65 versus 58, *P* < 0.001) with more women (50.0 versus 23.6%, *P* < 0.001), Hispanic ethnicity (40.5 versus 17.7%, *P* = 0.001), obesity (69.0 versus 29.9%, *P* < 0.001), diabetes mellitus (59.5 versus 27.8%, *P* < 0.01), insulin-dependence (23.8 versus 10.2%, *P* = 0.007), hyperlipidemia (40.5 versus 10.5, *P* < 0.001), and statin use (33.3 versus 5.3%, *P* < 0.001). Cumulative incidence of waitlist dropout at 2 y was 17.4% (95% confidence intervals, 7.7-30.4) for NAFLD HCC and 25.4% (95% confidence intervals, 21.9-29.0) for other HCC (*P* = 0.28). No difference in waitlist dropout or receipt of LT between NAFLD HCC and other HCC was found on regression analysis. Similarly, NAFLD and obesity, obesity alone, diabetes mellitus, insulin-dependence, hyperlipidemia, and statin use were not associated with waitlist outcomes. Finally, we observed no statistically significant difference in 5-y survival from HCC diagnosis between NAFLD HCC and other HCC (78.5% versus 66.9%, *P* = 0.9).

**Conclusions.:**

In our single-center cohort, we observed no difference in waitlist outcomes or survival in NAFLD HCC, although conclusions are limited by the small number of NAFLD-HCC patients. Notably, the inclusion of patients with obesity in the NAFLD-HCC group and stratification by individual metabolic factors also showed no difference in waitlist outcomes.

## INTRODUCTION

Primary liver cancer, of which the majority are hepatocellular carcinoma (HCC), is the seventh most common cancer worldwide and the third leading cause of cancer-related mortality.^1^ In the United States, the incidence of HCC has more than tripled over the last decade and liver cancer is currently the fastest growing cause of cancer death.^2-5^ With the increasing prevalence of metabolic syndrome and advances in treatments for viral hepatitis, nonalcoholic fatty liver disease (NAFLD) has become a leading etiology of chronic liver disease and HCC.^5,6^ NAFLD-associated HCC (NAFLD HCC) is already the most rapidly growing indication for liver transplantation (LT) in HCC patients in the United States,^5-7^ and, with a projected NAFLD prevalence of over 30% in the United States by 2030, there is expected to be a 137% increase in HCC incidence and 178% increase in liver deaths over the next decade.^8^

Several population studies have shown that patients with NAFLD HCC have worse outcomes when compared with patients with other etiologies of HCC (other HCC), specifically decreased probability of LT and decreased overall survival.^9,10^ NAFLD-HCC patients have risk factors independently associated with HCC development,^11-13^ more advanced stage at the time of HCC diagnosis^9^, and increased medical comorbidities, including cardiovascular disease.^10^ However, several studies have shown no difference in outcomes by etiology once patients undergo treatment with curative intent.^14-16^ In fact, patients with NAFLD HCC who undergo LT have been shown to have more favorable explant pathology^15^ and better posttransplant survival when found to be outside Milan criteria on explant.^16^ Possible explanations for this discrepancy are waitlist factors, such as increased dropout due to tumor progression, liver disease, or medical comorbidities, and prewaitlist factors, including differences in screening, diagnosis, and referral and listing for transplant.

A recent study using UNOS data of HCC patients on the waitlist for LT suggested that patients with NAFLD HCC were less likely to undergo LT,^17^ but the study did not include survival data, reason for waitlist dropout, or data on metabolic comorbidities. Using a cohort of HCC patients listed for LT, we aimed to further evaluate this discrepancy by evaluating risk of waitlist dropout, probability of LT, association with metabolic risk factors, and overall survival of patients with NAFLD HCC compared with other HCC.

## MATERIALS AND METHODS

### Study Design and Patient Population

This retrospective cohort study included 631 patients aged 18 y or older listed for LT with initial HCC model for end-stage liver disease (MELD) exception granted from January 2004 through December 2013 at University of California, San Francisco. The end date was chosen to allow for adequate waitlist and post-LT follow-up. Patients were excluded if their tumor burden was beyond Milan criteria (1 tumor 2–5 cm, 2–3 tumors ≤3 cm) at any point before transplant, even if downstaged successfully into Milan criteria given that this approach to LT was not standardized nationally during this study period. Etiology of liver disease was collected from listing diagnosis in UNOS and confirmed through chart review. NAFLD-HCC patients were compared with patients with all other etiologies of HCC (other HCC), and NAFLD HCC was defined as patients with HCC in the setting of metabolic risk factors (obesity, diabetes, hyperlipidemia) and the absence of other etiologies of liver disease. Other collected variables included age, sex, race/ethnicity, body mass index (BMI), presence of diabetes mellitus and hyperlipidemia, insulin-dependence, statin use, size and number of tumors at the time of priority listing, number, and type of local-regional therapies (LRTs) received, alpha fetoprotein (AFP) level at listing and transplant if applicable, MELD-Na score at listing, and reason for waitlist dropout or explant histopathologic data if applicable.

### Outcomes and Statistical Analysis

Primary outcome was risk of waitlist dropout, defined as HCC progression or liver death. Secondary outcomes included (1) probability of LT defined as a deceased donor and living donor LT; (2) intention-to-treat survival from time of HCC diagnosis defined as a waitlist or post-LT death; (3) post-LT HCC recurrence defined as presence of LIRADS 5 or biopsy-proven liver lesion, macrovascular invasion, or metastasis on post-LT surveillance imaging; and (4) post-LT patient survival with patient death as the event. Patients were stratified by etiology of liver disease, and clinical characteristics were compared with Pearson’s chi-square and Kruskal-Wallis tests. The cumulative incidence and 95% confidence intervals (CIs) for dropout and LT were calculated from date of listing while accounting for competing risks and stratified by liver disease etiology. For the primary outcome of dropout, LT was considered a competing event. For the secondary outcome of LT, dropouts due to liver-related death or HCC tumor progression were considered competing for events. Patients remaining on the waitlist or removed for other reasons were censored at the last known date on the waitlist.

Intention-to-treat survival and post-LT events, including HCC recurrence and post-LT survival, were estimated using Kaplan-Meier methods and compared by etiology of HCC using the log-rank test. For the intention-to-treat analysis, patients were followed from the date of HCC diagnosis to waitlist death or post-LT death. For HCC recurrence, patients were followed from the date of LT to HCC recurrence with patients censored at the date of death or last follow-up. For post-LT survival, patients were followed from LT date with those remaining alive censored at last follow-up. Univariable and multivariable subdistribution hazard ratios (HRs) and 95% CIs were estimated separately for factors associated with (1) waitlist dropout and (2) LT via Fine and Gray competing risk regression. Factors with a univariable *P* value <0.1 were evaluated in the multivariable analysis. The final multivariable models were selected by backward elimination, *P* for removal >0.05, while retaining the primary factor of interest in the model regardless of statistical significance. Statistical analyses were performed with SAS, version 9.4 (SAS Institute Inc., Cary, NC) and Stata/IC 14.2 (StataCorp, College Station, TX). This study was approved by the University of California, San Francisco Committee for Human Research.

## RESULTS

### Cohort Characteristics

Baseline patient characteristics for the 631 patients included in the study are summarized in Table 1. Forty-two patients (6.7%) with NAFLD HCC were compared with 589 patients (93.3%) with other HCC, including hepatitis C virus (63.1%), hepatitis B virus (20.9%), alcohol use (6.3%), and autoimmune disease (2.2%). Patients with NAFLD HCC were older (62 versus 58 y, *P* < 0.001) with a higher percentage of women (50.0% versus 23.6%, *P*  < 0.001) and Hispanic ethnicity (40.5% versus 17.7%, *P* = 0.001). They were also more likely to have components of metabolic syndrome, namely higher prevalence of obesity as defined by BMI ≥ 30 (69.0% versus 29.9%, *P* < 0.001) with median BMI 32.7 versus 27.1 (*P* < 0.001), diabetes (59.5% versus 27.8%, *P* < 0.001), insulin-dependence (23.8 versus 10.2%, *P* = 0.007), and hyperlipidemia (40..5% versus 10.5%, *P* < 0.001) with statin use (33.3 versus 5.3%, *P* < 0.001). There was no difference in listing MELD-Na or blood type, although NAFLD-HCC patients had lower listing AFP than other HCC (8 versus 14 ng/mL, *P* = 0.03). The majority of patients had a single lesion at time of listing with comparable overall tumor burden (*P* = 0.38) and number of LRT (*P* = 0.30) between patients with NAFLD HCC and other HCC.

**Table 1.** Waitlist patient and tumor characteristics

	Total (n = 631)	NAFLD HCC (n = 42)	Other HCC (n = 589)	*P*
**Median age (IQR**)	59 (54–63)	65 (61–67)	58 (54–62)	<0.001
**Female gender (%**)	160 (25.3)	21 (50.0)	139 (23.6)	<0.001
**Race/ethnicity (%**)				
** **Caucasian	277 (43.9)	18 (42.9)	259 (44.0)	0.001
** **Asian	169 (26.8)	5 (11.9)	164 (27.8)	
** **Hispanic	121 (19.1)	17 (40.5)	104 (17.7)	
** **Black	42 (6.7)	0	42 (7.1)	
**Blood type (%**)				0.11
** **A	221 (35.0)	8 (19.0)	213 (36.2)	
** **B	90 (14.3)	6 (14.3)	84 (14.3)	
** **O	295 (46.8)	25 (59.5)	270 (45.8)	
** **AB	25 (4.0)	3 (7.1)	22 (3.7)	
**Obesity (BMI **≥** 30**)	205 (32.4)	29 (69.0)	176 (29.9)	<0.001
** **<25	174 (28.9)	2 (4.9)	172 (30.7)	<0.001
** **25–29.9	223 (37.0)	10 (24.4)	213 (38.0)	
** **30–34.0	135 (22.4)	17 (41.5)	118 (21.0)	
** **≥35	70 (11.6)	12 (29.3)	58 (10.3)	
**Diabetes mellitus**	189 (30.0)	25 (59.5)	164 (27.8)	<0.001
** **Insulin-dependent	70 (11.1)	10 (23.8)	60 (10.2)	0.007
** **Median A1c (IQR)	6.9 (6.0–7.4)	6.8 (6.3–7.2)	6.9 (6.0–7.4)	0.77
**Hyperlipidemia**	79 (12.5)	17 (40.5)	62 (10.5)	<0.001
** **On statin	45 (7.1)	14 (33.3)	31 (5.3)	<0.001
**Median MELD-Na (IQR**)	11 (8–14)	11 (9–14)	10 (8–14)	0.54
**Median AFP (ng/mL) (IQR**)	13 (6–63)	8 (4–24)	14 (6–69)	0.03
**Tumor burden at listing (%**)				
** **1 lesion 2–3 cm	286 (45.3)	16 (38.1)	270 (45.8)	0.38
** **1 lesion 3.1–5 cm	181 (28.7)	17 (40.5)	164 (27.8)	
** **2 lesions	123 (19.5)	7 (16.7)	116 (19.7)	
** **3 lesions	41 (6.5)	2 (4.8)	39 (6.6)	
**Number of LRTs (%**)				
** **0–1	262 (41.5)	21 (50.0)	243 (41.3)	0.30
** **2–3	284 (45.0)	19 (45.2)	255 (43.3)	
** **>3	93 (14.7)	2 (4.7)	91 (15.4)	

AFP, alpha fetoprotein; BMI, body mass index; HCC, hepatocellular carcinoma; IQR, interquartile range; LRT, local-regional treatment; MELD-Na, model for end-stage liver disease with sodium; NAFLD HCC, hepatocellular carcinoma due to nonalcoholic fatty liver disease; other HCC, HCC not due to NAFLD.

### Outcomes on the Waiting List

**FIGURE 1.**
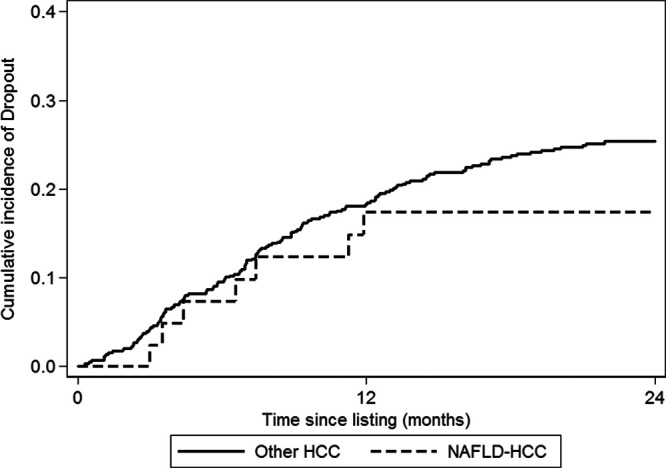
Risk of waitlist dropout in patients with NAFLD HCC compared with other HCC. NAFLD HCC, hepatocellular carcinoma due to nonalcoholic fatty liver disease; other HCC, patients with other etiologies of HCC.

Of 631 waitlisted patients, 155 patients (24.5%) dropped out because of tumor progression or death (Table 2), including 7 of 42 NAFLD-HCC patients (16.7%) and 148 of 589 other-HCC patients (25.1%). Another 46 patients were censored at the time of waitlist dropout for other factors precluding LT, including 22 patients with psychosocial issues (1 NAFLD-HCC patient), 19 patients with other medical comorbidities (2 NAFLD-HCC patients), and 5 patients who ultimately declined LT (no NAFLD-HCC patients). The cumulative incidence of dropout due to tumor progression or death was 24.8% (95% CI, 21.5-28.3%) at 2 y from listing 17.4% (95% CI, 7.7-30.4%) for NAFLD-HCC patients and 25.4% (95% CI, 21.9-29.0%) for other-HCC patients (Figure 1, *P* = 0.28). Median time to dropout was 7.4 mo (interquartile range, 3.6–12.4) overall and did not differ between NAFLD HCC and other HCC.

**Table 2.** Outcomes on liver transplant waitlist

	Overall (n = 631)	NAFLD HCC (n = 42)	Other HCC (n = 589)	*P*
**Waitlist outcome (%**)				
** **Liver transplant	425 (67.4)	32 (76.2)	393 (66.7)	0.65
** **Waitlist dropout^a^	155 (24.5)	7 (16.7)	148 (25.1)	
** **Other dropout	46 (7.3)	3 (7.1)	43 (7.3)	
** **Active on waitlist	5 (0.8)	0 (0)	5 (0.8)	
**Median time to dropout, mo (IQR**)	7.4 (3.6–12.4)	6.6 (3.5–11.3)	7.4 (3.7–12.4)	0.53
**Median time to transplant, mo (IQR**)	10.7 (6.6–15.8)	12.4 (6.9–16.1)	10.7 (6.6–15.8)	0.69
**Median survival from listing, mo (IQR**)	50.3 (18.6–90.8)	58.1 (38.0–88.1)	49.1 (17.8–90.9)	0.44
**Median AFP (IQR**)				
** **At transplant	7 (4–24)	6 (4–29)	8 (4–24)	0.40
**Pathological stage on explant (%**)				
** **No viable tumor	169 (40.0)	10 (31.3)	159 (40.6)	0.60
** **Within Milan	183 (43.2)	16 (50.0)	165 (42.1)	
** **Outside Milan	63 (14.9)	4 (12.5)	59 (15.1)	
** **Invasion of large vessels	1 (0.2)	0	1 (0.3)	
** **Metastatic disease	6 (1.4)	0	6 (1.5)	
**Explant grade of differentiation (%**)				
** **Complete necrosis	170 (40.1)	10 (31.3)	160 (40.8)	0.71
** **Well-differentiated	90 (21.2)	8 (25.0)	82 (20.9)	
** **Moderate differentiated	139 (32.8)	12 (37.5)	127 (32.4)	
** **Poorly differentiated	25 (5.9)	2 (6.3)	23 (5.9)	
**Microvascular invasion on explant (%**)	21 (5.0)	2 (6.3)	19 (4.8)	0.67

^a^Defined as dropout due to liver-related death (1 NAFLD-HCC patient, 42 other-HCC patients) and HCC progression (6 NAFLD-HCC patients, 106 other-HCC patients).

AFP, alpha fetoprotein; HCC, hepatocellular carcinoma; IQR, interquartile range; NAFLD HCC, hepatocellular carcinoma due to nonalcoholic fatty liver disease; other HCC, HCC not due to NAFLD.

**FIGURE 2.**
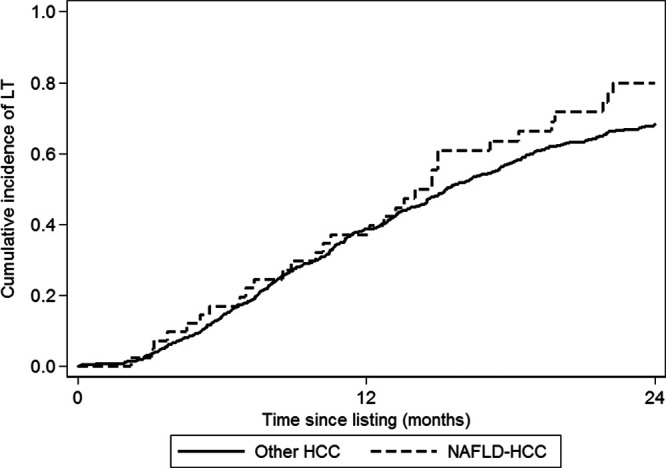
Probability of liver transplant in patients with NAFLD HCC compared with other HCC. NAFLD HCC, hepatocellular carcinoma due to nonalcoholic fatty liver disease; other HCC, patients with other etiologies of HCC.

Of the 631 waitlisted patients, 425 patients (67.4%) underwent LT (Table 2), including 32 of 42 NAFLD-HCC patients (76.2%) and 393 of 589 other-HCC patients (66.7%). From time of listing with HCC MELD exception, the overall cumulative incidence of LT was 69.1% (95% CI, 65.2-72.7%) at 2 y with 79.9% (95% CI, 63.7-89.4%) for NAFLD-HCC patients and 68.3% (95% CI, 64.3-72.1%) for other-HCC patients (Figure 2, *P* = 0.16). Two patients underwent living donor LT, neither of which had NAFLD HCC. Median time to LT was 10.7 mo overall (interquartile range, 6.6–15.8 mo) and did not differ between NAFLD HCC and other HCC. In terms of liver explant pathology, there was no difference in grade of differentiation, microvascular invasion (6.3 versus 5.0%, *P* = 0.67), or pathological stage (12.5% versus 14.9% outside of Milan, *P* = 0.60).

Given the relatively low number of NAFLD-HCC patients, patients with NAFLD and obesity (NAFLD+obese HCC) were compared with patients without NAFLD or obesity (non-NAFLD + nonobese HCC). Of 631 patients on the LT waitlist, BMI data were available for 603 patients, including 218 patients with NAFLD + obese HCC (36.2%) and 385 patients with non-NAFLD + nonobese HCC (63.8%) (Table S1, SDC, http://links.lww.com/TXD/A284). NAFLD+obese-HCC cohort included a higher percentage of women (30.7% versus 21.6%, *P* = 0.01) and Hispanic ethnicity (27.5% versus 13.8%, *P* < 0.001). They were also more likely to have other components of metabolic syndrome, namely higher prevalence of diabetes (35.3% versus 27.0%, *P* = 0.03), hyperlipidemia (16.5% versus 10.1%, *P* = 0.02), and statin use (11.0% versus 4.9%, *P* = 0.01). There was no difference in age, blood type, insulin-dependence, listing AFP, or listing tumor burden, although NAFLD+obese-HCC patients had higher listing MELD-Na (12 versus 10, *P* < 0.001) and fewer LRT (*P* = 0.03).

Of these 603 patients, 144 patients (23.9%) dropped out because of tumor progression or death (Table S2, SDC, http://links.lww.com/TXD/A284), including 50 of 218 NAFLD+obese-HCC patients (22.9%) and 94 of 385 non-NAFLD+nonobese-HCC patients (24.4%). The cumulative incidence of dropout at 2 y was 24.7% (95% CI, 20.4-29.2%) for NAFLD+obese-HCC patients and 23.2% (95% CI, 17.7-29.1%) for patients with non-NAFLD+nonobese HCC. Four hundred and nine of 603 patients (67.8%) underwent LT, including 153 of 218 NAFLD+obese-HCC patients (70.2%) and 256 of 385 NAFLD+nonobese-HCC patients (66.5%). The cumulative incidence of LT at 2 y was 67.8% (95% CI, 62.7-72.4%) for NAFLD+obese-HCC patients and 72.6% (95% CI, 65.9-78.1%) for patients with non-NAFLD+nonobese HCC. Regarding explant pathology, there were fewer patients with complete necrosis and more with poorly differentiated tumors on pathology review (34.0% versus 44.7% and 9.2% versus 4.3%, respectively, *P* = 0.03) in the NAFLD+obese-HCC patients. There was no difference in AFP at transplant nor microvascular invasion on explant pathology.

### Outcomes Analysis

On univariable analysis, there was no statistically significant difference in risk of waitlist dropout between NAFLD-HCC patients and those with other HCC (hazard ratios [HRs], 0.65; 95% CI, 0.30-1.41; *P* = 0.28, Table 3), although notably our analysis was underpowered because of only 42 NAFLD-HCC patients. Factors that were associated with waitlist dropout included race/ethnicity (other versus white: HR, 2.08; 95% CI, 1.07-4.05; *P* = 0.03), tumor burden (1 tumor 2–3 cm: HR, 2.21; 95% CI, 1.51-3.23; *P* < 0.001; 3 tumors: HR, 3.50; 95% CI, 2.03-6.02; *P* < 0.001), AFP at listing ≥20 ng/mL (HR, 0.52; 95% CI, 0.38-0.71; *P* < 0.001), and number of LRT (HR, 1.68; 95% CI, 1.11-2.53; *P* = 0.01). On multivariable analysis including NAFLD HCC, race/ethnicity, tumor burden, MELD-Na, and AFP at listing, and number of LRT, there was no statistically significant difference between NAFLD-HCC and other-HCC patients (HR, 0.65; 95% CI, 0.30-1.41; *P* = 0.28), while factors that were associated with waitlist dropout included tumor burden (1 lesion 3–5 cm: HR, 2.25; 95% CI, 1.51-3.23; *P* < 0.001 or 3 lesions: HR, 3.00; 95% CI, 1.70-5.30; *P* < 0.001), MELD-Na at listing ≥15 (HR, 1.55; 95% CI, 1.05-2.28; *P* = 0.03), and AFP at listing ≥20 ng/mL (HR, 1.88; 95% CI, 1.36-2.60; *P* < 0.001). NAFLD+obese HCC, obesity, diabetes, insulin-dependence, hyperlipidemia, and statin use were not associated with risk of waitlist dropout (Table 3).

**Table 3.** Univariate and multivariable analysis of predictors of waitlist dropout by Cox competing risk regression

Variable	UV HR (95% CI)	*P*	MV HR (95% CI)	*P*
**NAFLD HCC**	0.65 (0.30-1.41)	0.28	0.65 (0.30-1.41)	0.28
**NAFLD+obese HCC**	0.95 (0.67-1.34)	0.77	0.92 (0.65-1.31)	0.65
**Age at listing**	1.00 (0.98-1.02)	0.90		
**Female gender**	1.04 (0.73-1.50)	0.81		
**Race/ethnicity**				
** **Caucasian	1.00			
** **Asian	0.72 (0.47-1.08)	0.12		
** **Hispanic	0.93 (0.6-1.45)	0.75		
** **Black	1.62(0.94-2.79)	0.08		
** **Other	2.08 (1.07-4.05)	0.03		
**Obesity** (BMI ≥ 30)	0.95 (0.67-1.35)	0.77		
** **<25	1.00			
** **25–29.9	0.79 (0.53-1.17)	0.23		
** **30–34.0	0.87 (0.56-1.36)	0.54		
** **≥35	0.77 (0.43-1.37)	0.37		
**Diabetes mellitus**	0.75 (0.53-1.08)	0.13		
** **Insulin-dependent	1.15 (0.71-1.84)	0.57		
**Hyperlipidemia**	1.23 (0.79-1.91)	0.35		
** **Statin use	1.19 (0.68-2.09)	0.54		
**Blood type**				
** **AB+B vs A+O	0.70 (0.44-1.12)	0.14		
**Tumor burden**				
** **1 lesion 2–3 cm	1.00 (ref)	<0.001	1.00 (ref)	<0.001
** **1 lesion 3.1–5 cm	2.21 (1.51-3.23)	0.11	2.25 (1.53-3.29)	0.09
** **2 lesions	1.45 (0.92-2.29)	<0.001	1.49 (0.94-2.37)	<0.001
** **3 lesions	3.5 (2.03-6.02)		2.97 (1.68-5.26)	
**MELD-Na at listing** ≥**15**	2.79 (1.35-5.76)	0.006	1.55 (1.05-2.28)	0.03
**AFP at listing** ≥**20 ng/mL**	0.52 (0.38-0.71)	<0.001	1.88 (1.36-2.60)	<0.001
**Number of LRT**				
** **0–1	1.00 (ref)	0.49		
** **2–3	1.14 (0.79-1.63)	0.01		
** **>3	1.68 (1.11-2.53)			

AFP, alpha fetoprotein; BMI, body mass index; CI, confidence interval; HCC, hepatocellular carcinoma; HR, hazard ratio; LRT, local-regional treatment; MELD-Na, model for end-stage liver disease with sodium; MV, multivariable; NAFLD HCC. hepatocellular cancer due to nonalcoholic fatty liver disease; UV, univariable.

Similarly, there was no statistically significant difference in probability of LT in NAFLD-HCC patients compared with other HCC in univariable analysis (HR, 1.27; 95% CI, 0.91-1.77; *P* = 0.16), again limited by small sample size. Factors that were associated with probability of LT included HgbA1c (continuous variable: HR, 0.82; 95% CI, 0.68-0.99; *P* = 0.04), blood type (AB+B versus A+O: HR, 1.73; 95% CI, 1.30-2.30; *P* < 0.001), tumor burden (1 tumor 3–5 cm: HR, 0.64; 95% CI, 0.51-0.81; *P* < 0.001; 3 tumors: HR, 0.48; 95% CI, 0.30-0.76; *P* = 0.002), MELD-Na at listing ≥15 (HR, 2.99; 95% CI, 1.73-5.17; *P* < 0.001), AFP at listing ≥20 ng/mL (HR, 1.38; 95% CI, 1.13-1.68; *P* = 0.001), and number of LRT (LRT 2-3: HR, 0.77; 95% CI, 0.62-0.95; *P* = 0.01; LRT >3: HR, 0.47; 95% CI, 0.36-0.62; *P* < 0.001). On multivariable analysis including NAFLD HCC, obesity, insulin-dependence, race/ethnicity, blood type, tumor burden, AFP and MELD-Na at listing, and number of LRT, there was still no difference between NAFLD-HCC and other-HCC patients (HR, 1.25; 95% CI, 0.89-1.76; *P* = 0.20). HgbA1c was not included because the sample size was too small. Independent predictors associated with probability of LT were MELD-Na listing ≥15, decreased tumor burden, fewer LRT, and blood type AB or B. NAFLD+obese HCC, obesity, diabetes, insulin-dependence, hyperlipidemia, and statin use were not associated with probability of LT.

**FIGURE 3.**
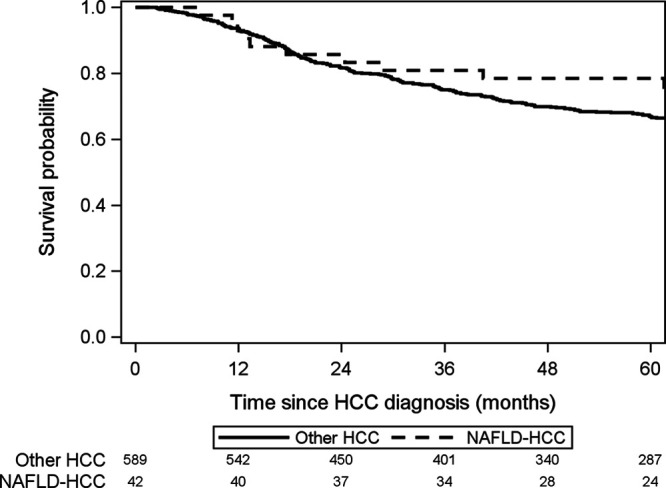
Intention-to-treat survival from time of HCC diagnosis for patients with NAFLD HCC compared with other HCC. HCC, hepatocellular carcinoma; NAFLD HCC, hepatocellular carcinoma due to nonalcoholic fatty liver disease; other HCC, patients with other etiologies of HCC.

**FIGURE 4.**
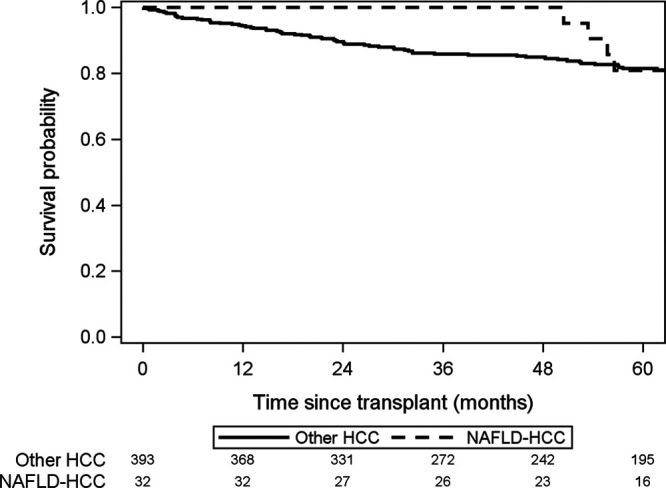
Probability of survival after liver transplant for patients with NAFLD HCC compared with other HCC. NAFLD HCC, hepatocellular carcinoma due to nonalcoholic fatty liver disease; other HCC, patients with other etiologies of HCC.

**FIGURE 5.**
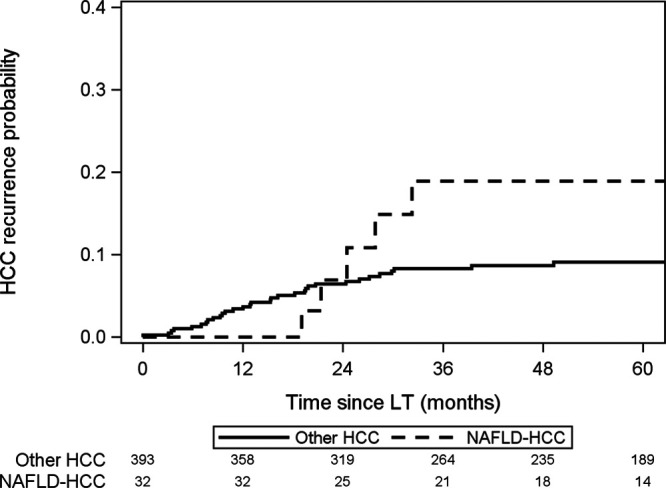
Probability of HCC recurrence after liver transplant for NAFLD HCC compared with other HCC. NAFLD HCC, hepatocellular carcinoma due to nonalcoholic fatty liver disease; other HCC, patients with other etiologies of HCC.

Overall, 5-y intention-to-treat survival from time of HCC diagnosis was 67.6% (95% CI, 63.7-71.3%). There was no statistically significant difference in 5-y intention-to-treat survival (78.5% versus 66.9%; 95% CI, 62.7-70.7% versus 62.7-88.2%; *P* = 0.91; Figure 3) or post-LT survival (81.0% versus 81.4%; 95% CI, 56.9-92.4% versus 76.8-95.2%; *P* = 0.97; Figure 4) between NAFLD HCC and other HCC. Of the 425 patients who underwent LT, only 39 patients had HCC recurrence at 5 y post-LT, including 5 NAFLD-HCC and 34 other-HCC patients (18.9% versus 9.1%, respectively; *P* = 0.21; Figure 5). Similarly, there was no statistically significant difference in post-LT HCC recurrence in NAFLD+obese-HCC patients compared with non-NAFLD+nonobese-HCC patients (16/153 patients, 10.4% versus 20/256 patients, 7.8%; *P* = 0.37).

## DISCUSSION

NAFLD is already among the leading etiologies of HCC and is the most rapidly increasing indication for HCC-related LT in the United States because of rising prevalence of obesity, diabetes, and metabolic syndrome.^3-8^ Prior studies of HCC populations reported decreased overall survival in patients with NAFLD HCC compared with other HCC in the setting of more advanced tumor stage at diagnosis, increased medical comorbidities, and risk factors independently associated with HCC development.^9-13^ However, our data suggest that, among HCC patients selected and listed for LT at our single large transplant center, there may be no difference in waitlist dropout, LT, or survival between patients with NAFLD HCC and patients with other HCC.

In our study of 631 patients with HCC listed for transplant, 67.4% of patients underwent LT and 24.5% of patients dropped out from waitlist before receiving transplant with no statistically significant difference found in the probability of LT or waitlist dropout between patients with NAFLD HCC and those with other HCC. Additionally, both groups had similar tumor burden at listing, patients underwent similar numbers of LRT, and, in LT recipients, there was no significant difference in explant pathology characteristics. Finally, patients with NALFD HCC also had similar intention-to-treat survival and post-LT HCC recurrence as patients with other etiologies of HCC. Although we are limited in drawing definitive conclusions from this data because of only 42 NAFLD-HCC patients, there was also no difference in waitlist outcomes by NAFLD+obese HCC nor individual metabolic risk factors.

Several studies have also shown no difference in HCC outcomes by etiology after treatment with curative intent.^14-16^ In fact, there is evidence to suggest potentially better outcomes after curative treatment in patients with NAFLD HCC: more favorable explant pathology in study of UNOS patients^15^ decreased HCC recurrence in patients found to be beyond Milan criteria on explant^16^ and even improved overall survival after curative treatment in 1 study.^14^ Our study also suggests there is also no difference in waitlist outcomes for NAFLD-HCC patients, including waitlist dropout due to HCC progression and overall survival from time of listing. Taken all together, these findings argue against inherently more aggressive tumor biology or increased morbidity or mortality from other comorbidities in NAFLD HCC among patients listed for LT or undergoing other curative therapies.

In our cohort, 32% of patients were obese, 30% had diabetes, and 13% hyperlipidemia with significantly higher prevalence of these metabolic factors in NAFLD-HCC patients compared with other-HCC patients. Both obesity and diabetes have been associated independently with risk of HCC,^11-13^ and increasing number of metabolic comorbidities (obesity, diabetes, hyperlipidemia, hypertension) have been associated with increasing HCC risk with the presence of all 4 factors carrying an over 8-fold increased HCC risk.^13^ Additionally, a recently developed risk stratification model for HCC risk in NAFLD-related cirrhosis includes diabetes and BMI.^18^ However, there is limited and conflicting data on how these metabolic factors affect outcomes in HCC patients.

We found no association between obesity and the probability of LT or waitlist dropout, and, whereas patients with NAFLD+obese HCC did have worse explant pathology by some measures, including less complete necrosis and more poorly differentiation tumors, there was no difference in microvascular invasion or post-LT HCC recurrence. A metaanalysis of 9 studies found that obesity (BMI ≥ 30) but not being overweight (BMI ≥ 25) was associated with higher HCC-related mortality than patients with normal BMI.^19^ Interestingly, the association with obesity and worse outcomes was only true for Western populations (ie, non-Asian) and was seen to a greater degree in obese men. However, the authors found moderate risk of bias in these studies as BMI was self-reported in the majority of studies and none of the studies controlled for cirrhosis, HCC, etiology, HCC stage, or treatment modalities. Another metaanalysis of 14 studies evaluating HCC patients undergoing surgical resection of HCC found no association between obesity and disease-free or overall survival.^20^ Similarly, the limited data for patients awaiting LT is conflicting.^21-24^ Potential reasons for the discrepancies are different BMI cutoffs, the inclusion of underweight patients when evaluated as dichotomous variable (ie, BMI  < 30 includes BMI < 18), and the inherent limitations of using BMI to assess for obesity in patients with liver disease. Notably, our study did not include patients with BMI ≥ 50 (contraindication to LT at our center), and BMI was not adjusted for volume overload, although median MELD and Child-Pugh score were 11 and 7, respectively, at listing, suggest a well-compensated cohort. Recent studies using other measures of obesity (increased visceral or subcutaneous fat) and malnutrition (loss of skeletal muscle mass) have more consistently found an association with increased HCC recurrence and higher mortality.^25,26^

We also found no association between diabetes and the probability of LT or waitlist dropout. A metaanalysis of 21 studies concluded that diabetes was independently associated with decreased disease-free and overall survival^27^; however, like the obesity data, there was significant heterogeneity in these studies, and 9 of the studies evaluated patients undergoing hepatic resection only. One possible explanation for the discrepancy between our findings and the metaanalysis is the variable duration and control of diabetes in different patient populations with patients awaiting transplant more likely to have controlled diabetes because of close monitoring, which is supported by our finding that there was no difference in median A1c in diabetic patients with NAFLD-HCC compared with other etiology of HCC. However, 3 studies of patients awaiting or having undergone LT published since the metaanalysis also reported worse outcomes in HCC patients with diabetes.^21,28,29^ A recent study showed that diabetes was a predictor of decreased survival in the interferon era but not the direct-acting antiviral era, but the reasons for this are unclear.^30^ Finally, it also appears that BCLC stage is important, as Su et al reported lower survival rates in HCC patients with diabetes and BCLC stage 0, A, and B, but no difference in survival seen in stages C and D.^31,32^

For hyperlipidemia, the data are even more challenging to interpret as there are fewer studies with varying definitions of hyperlipidemia/dyslipidemia, the use of serum cholesterol values from postcirrhotic time points, and conflicting results.^32-34^ In our study, we used a chart diagnosis of hyperlipidemia (and not cholesterol levels) and also assessed statin use, and we found no difference in waitlist outcomes.

Taken all together, our data suggest that the decreased probability of LT and overall survival seen in NAFLD-HCC patients in population studies may be due to prewaitlist factors. This is further supported by the observation that NAFLD patients are more likely to be diagnosed with HCC at advanced stages and therefore not candidates for curative treatments.^9^ Possible explanations for this include biological factors, such as more aggressive tumor biology, but also systematic factors, such as less referral to specialists,^35^ decreased HCC screening rates,^35,36^ and screening methods that are less effective in this population.^37,38^ Even in NAFLD-HCC patients eligible for curative therapies, increased comorbidities, and relatively preserved liver function may lead to a preference for LRT over referral for transplant evaluation. Similarly, NAFLD-HCC patients undergoing LT evaluation are less likely to be listed than other HCC patients due to increased medical comorbidities, although these patients still die from their liver disease.^39^ Notably, our transplant center generally requires adequate control of metabolic comorbidities (preferred BMI  < 40, BMI ≥ 50 is an absolute contraindication, median Hgb A1c 6.9 in our cohort) and an intensive cardiac risk stratification for patients with multiple metabolic risk factors (ie, left heart catheterization rather than stress test) before listing, which leads to a highly selected cohort of NAFLD HCC that are eligible for listing. In contrast to our study, Young et al found that NAFLD-HCC patients listed for LT through UNOS were less likely to receive LT, which may be due to potential differences in national practices regarding prelisting metabolic work-up; however, Young et al^17^ also showed that NAFLD-HCC patients were less likely to received MELD exception points for HCC, suggesting that nonbiologic factors are also likely contributing to this discrepancy.

There are several limitations to our study, including the retrospective design. The study population was derived from a single transplant center and as such may not be generalizable to the country at large; however, this was a large cohort at a high-volume transplant center in a long wait time region allowing us to evaluate waitlist dropout and more granular data than national studies, such as metabolic risk factors. Our study population was very diverse with 27% Asian patients, 19% Hispanic patients, and 21% hepatitis B virus-associated HCC, which is consistent with our regional population though may not be generalizable to other populations. Notably, 41% of NAFLD-HCC patients were Hispanic, highlighting a population at particularly high risk for NAFLD and NAFLD-HCC. The study population only included 7% NAFLD-HCC patients, which is consistent with other published data during the same time period of the study; however, this did result in few events within the NAFLD-HCC group for waitlist dropout limiting our ability to detect statistical differences by etiology. We did, however, have a high prevalence of metabolic risk factors, which likely captured other patients with NAFLD or combined NAFLD and other etiology, and these metabolic factors were also not associated with risk of waitlist dropout or probability of LT. Similarly, when NAFLD+obese-HCC patients were combined in a secondary analysis, there was no statistically significant difference in waitlist dropout or LT compared with non-NAFLD+nonobese-HCC patients.

In summary, among HCC patients within Milan criteria and listed for LT, we observed no difference in waitlist dropout, LT, intention-to-treat survival, or post-LT HCC recurrence between patients with NAFLD HCC and patients with other HCC despite significant differences in demographics and metabolic risk factors. Similarly, the presence of obesity, diabetes, or hyperlipidemia did not impact waitlist outcomes. Our data argue against inherently more aggressive tumor biology in NAFLD HCC among patients listed for transplant, which is especially important given NAFLD HCC is the most rapidly rising indication for LT among HCC patients. Future studies are needed to further evaluate discrepancies between NAFLD HCC and other HCC with regards to screening methods, listing practices, prewaitlist tumor biology, and national waitlist outcomes.
